# Response Modality vs. Target Modality: Sensory Transformations and Comparisons in Cross-modal Slant Matching Tasks

**DOI:** 10.1038/s41598-018-29375-w

**Published:** 2018-07-23

**Authors:** Juan Liu, Hiroshi Ando

**Affiliations:** Center for Information and Neural Networks (CiNet), National Institute of Information and Communications Technology (NICT) and Osaka University, Osaka, Japan

## Abstract

Humans constantly combine multi-sensory spatial information to successfully interact with objects in peripersonal space. Previous studies suggest that sensory inputs of different modalities are encoded in different reference frames. In cross-modal tasks where the target and response modalities are different, it is unclear which reference frame these multiple sensory signals are transformed to for comparison. The current study used a slant perception and parallelity paradigm to explore this issue. Participants perceived (either visually or haptically) the slant of a reference board and were asked to either adjust an invisible test board by hand manipulation or to adjust a visible test board through verbal instructions to be physically parallel to the reference board. We examined the patterns of constant error and variability of unimodal and cross-modal tasks with various reference slant angles at different reference/test locations. The results revealed that rather than a mixture of the patterns of unimodal conditions, the pattern in cross-modal conditions depended almost entirely on the response modality and was not substantially affected by the target modality. Deviations in haptic response conditions could be predicted by the locations of the reference and test board, whereas the reference slant angle was an important predictor in visual response conditions.

## Introduction

Humans use multi-sensory information when interacting with objects in peripersonal three-dimensional (3D) space. For example, a person can pick up a hat and put it on without looking, requiring only slight adjustment using a mirror. Similarly, a person may pick up a ribbon and tie a bow behind their back without difficulty. Using multiple modalities for motor planning in various locations is not trivial because different sensory inputs are encoded in different reference frames (RFs). Visual information is represented retinotopically^1^, relative to current gaze direction, while somatosensory information from arms and hands, which can be further divided into discrete modalities^2^ (e.g., tactile and proprioceptive sensation), is represented relative to the body^3^, shoulder, or hand^4^. Thus, sensory inputs gathered with various gaze directions and body postures are combined to form coherent representations of the whole space. To utilise available information for comparison and planning at different locations, some signals must be transformed within or between RFs.

Previous studies reported that sensory transformation can incur a cost, adding bias and variability^5,6^. It has been suggested that when unimodal comparison is possible, the central nervous system (CNS) tends to avoid performing unnecessary coordinate transformations that may add noise^7^. However, in cross-modal tasks, transformations between modalities are inevitable. A number of psychophysical and neuroimaging studies have used the reaching task paradigm to examine how multiple sensory signals are processed, and which RFs are used for motor planning. These studies have produced disparate results, variously indicating that the RF used in motor planning is retinotopic^1,8–11^, hand- or body-centred^12–14^, or a common representation^15–17^. Recent studies have reconciled these divergent data, demonstrating that if the task precludes the unimodal comparison of target and hand information, the CNS represents movement plans simultaneously in multiple RFs for task-dependent reweighting and optimal use of available sensory information^18,19^.

The reaching tasks used in these studies have involved several common restrictions. First, because most tasks were conducted using hand responses, somatosensory information is available the whole time. Several previous experiments^20,21^ relied on the conflict between visual and somatosensory feedback about the hand, emphasising the process of combining redundant information more than RF transformation. Second, the workspaces used in these tasks have typically been small, and located in front of participants. Moreover, visual and somatosensory representations have typically been related to the same area, with participants completing the task without body or head movement. Thus, the relationships among multimodal spatial representations for different areas of peripersonal space have not been systematically studied. Third, some experiments^12,13,18,22^ required participants to memorise the target, making it difficult to dissociate the role played by sensory transformation from that of the short-term memory buffer. An internal representation may be formed only in relation to the memory storage of the target, but this representation might not be needed when pointing directly toward an actual target^3,23^. Previous haptic studies reported that a 10-s delay between exploring the target and responding in a unimodal haptic task could increase the contribution of the allocentric RF^24^, involving different visual areas for visual imagery^25^.

Perception of orientation in 3D space provides an alternative experimental paradigm for exploring human strategies for processing sensory information. Psychophysical studies have reported that both visual and haptic perception of orientation are susceptible to systematic deviations in frontal peripersonal space^26,27^. For example, visual perception of geographical slant (hills) involves gross overestimation and asymmetrical bias away from horizontal and toward vertical, such that hills are typically perceived as steeper than they actually are^26,28^. Moreover, haptic perception of parallelity of bars shows an egocentric bias in the direction of local hand orientation (i.e., the right bar must be rotated clockwise relative to the left bar to be perceived as haptically parallel)^4,29–31^. A RF intermediate to an allocentric frame and a body-centred egocentric frame has been proposed for haptic parallelity tasks^27,29^. However, most of these studies focused on one or two kinds of modality conditions, such as haptic-only^4^, visuo-only^28^, visuo-haptic^26,32,33^ or haptic-visual^34^ tasks, and did not investigate the transformations and comparison among RFs.

In the current study, we sought to expand on previous studies by using a new slant perception and parallelity paradigm for investigating cross-modal RFs. In our experiment, participants perceived (either visually or haptically) a reference board (i.e., either a visual target or a haptic target) at various slant angles, and were asked to either rotate an invisible test board by hand manipulation (haptic response) or to adjust a visible test board by giving verbal instructions to the experimenter to rotate it (i.e., response based on visual information, referred to as “visual response” hereafter) until they judged that the test board was physically parallel to the reference board. The reference and test boards were placed at several locations in the midsagittal plane involving both frontal and rear peripersonal space. In haptic conditions, participants combined their proprioceptive and tactile sensations with motor efference copy to create a unified perception of the slant. The accuracy and precision of the responses were measured in the resulting four task conditions: (1) visual target – visual response (VV), (2) visual target – haptic response (VH), (3) haptic target – haptic response (HH), (4) haptic target – visual response (HV). We were able to examine situations in which haptic information was absent for responses (VV), and situations in which multisensory information was not redundant in the comparison (VH, HV). Apart from the VV condition, the reference and test boards could be perceived simultaneously by vision or the hands during the matching process, not requiring storage in short-term memory. Using this paradigm, we investigated direct comparisons in unimodal conditions and RF transformations in cross-modal conditions, identifying the rules governing the processing flow of cross-modal sensory transformation and comparison.

**Figure 1:**
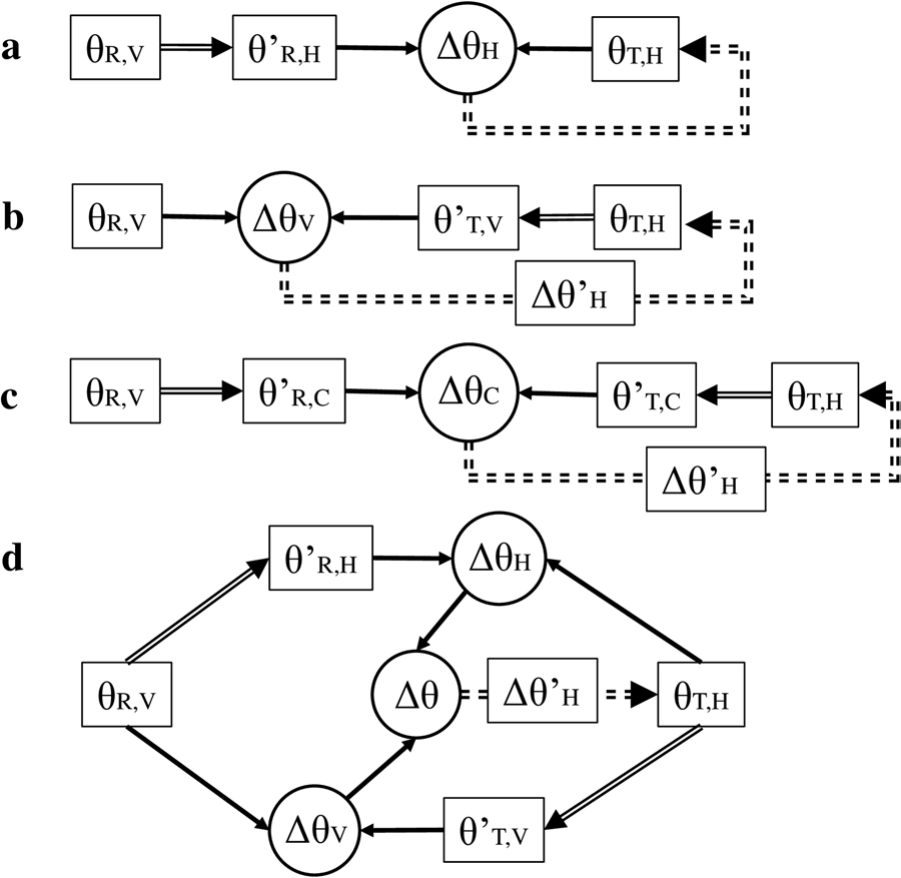
Four possible models for the use of visual (V) and haptic (H) information in a cross-modal matching task (VH condition). In each model, the slant of the reference surface (R) was perceived visually (θ_R,V_), and the slant of the test surface (T) was adjusted haptically (θ_T,H_). Comparisons of the slant angles of the two surfaces could be performed in the response modality (haptic RF in (**a**)), the target modality (visual RF in (**b**)), or a common RF (**c**) after transformations (double solid lines) of the information among visual, haptic or common RF. The result of the comparison could also be the combination (Δθ) of individual comparisons performed separately in each sensory modality to drive the response, as shown in (**d**). The double dashed line shows that the results of comparison were used for adjusting the slant angle of the test surface, so that Δθ_•_ = 0 (• ∈ {H, V, C}).

We assumed that in our unimodal conditions (VV and HH), no RF transformation between modalities would occur because the sensory modality directly comparable to the target was privileged, avoiding nonessential transformations that added noise^7,19^. Constant errors in slant matching at various test locations would thus reflect the features of comparison and transformation within the single modality. In the cross-modal conditions (VH and HV), four possible types of processing flow of comparisons could be performed (e.g., models for the VH condition, as shown in Fig. 1). The RF used for comparison could be that of the response modality, the target modality, a common RF, or the RFs of both modalities to produce a combination of individual comparisons.

## Results

**Figure 2:**
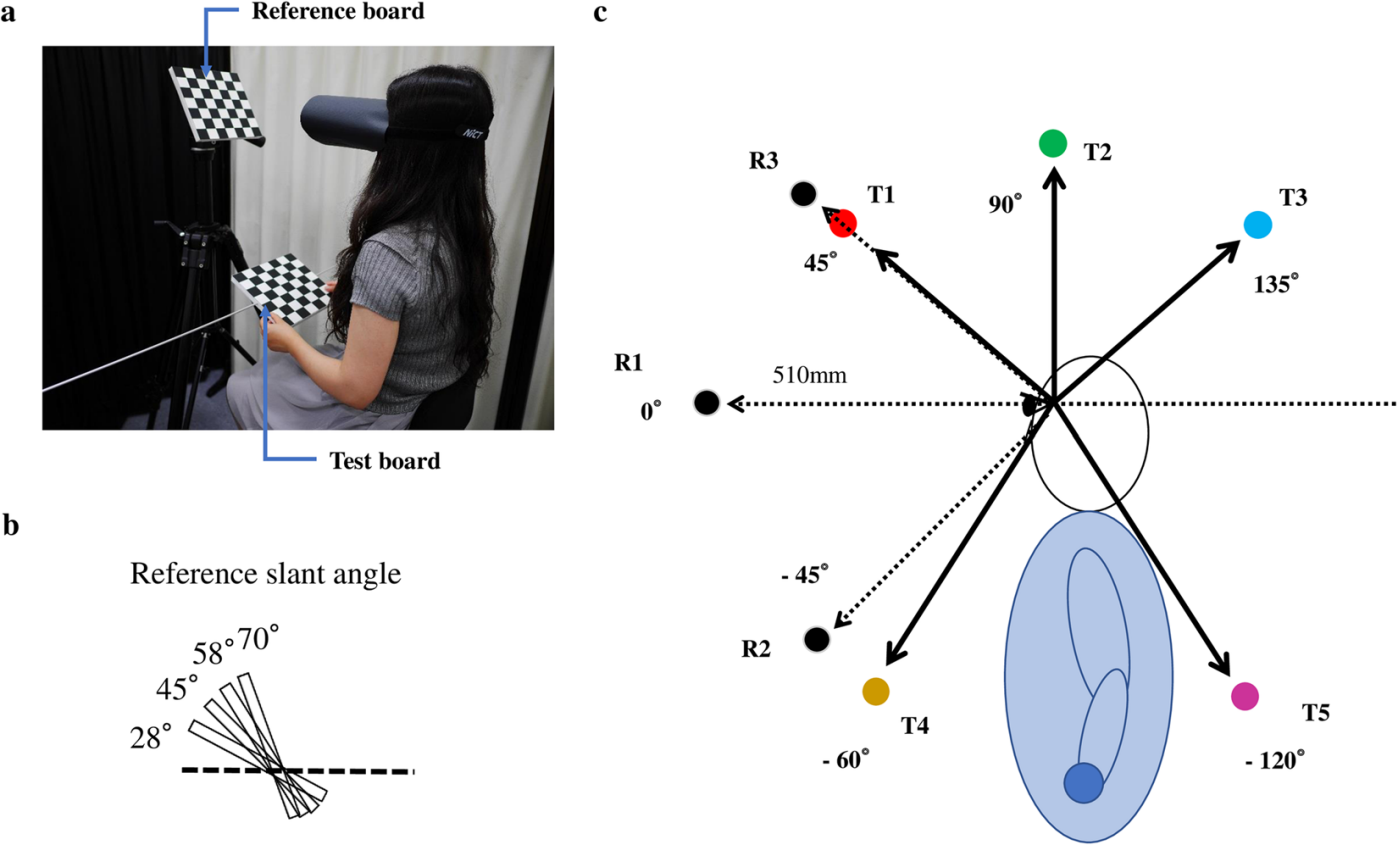
(**a**) Experimental setup (photographed by Hiroshi Ando). (**b**) The four slant angles of the reference board. (**c**) Reference locations (R1, R2, R3) and test locations (T1–T5). E.g., the setting in (**a**) is the VH modality condition with R1T4 location condition and 58° reference slant angle.

We examined error patterns and variability changes in slant matching tasks (Fig. 2a) at various reference/test locations in peripersonal space under different target/response modality conditions. By changing the reference location (R1, R2 and R3 in Fig. 2c), we investigated the influence of target modality and introduced more recognisable patterns into the bias. A less egocentric RF would result in smaller effects of the reference location condition. The four reference slant angles (Fig. 2b) were also used to provide more detail about the bias pattern. The test locations (T1–T5 in Fig. 2c) were varied in the midsagittal plane. Two pairs of reference/test locations were excluded because they were overlapping (R3/T1 [45°/45°]) or too close (R2/T4 [−45°/−60°]). Thus, only 13 pairs of reference/test locations were used in the experiment. There were 208 conditions in total (13 reference-test location pairs × 4 slant angles × 4 modality conditions) and each participant underwent one trial for each condition. We compared differences in constant error and variability then identified the most important factors for prediction of slant matching results under different modality conditions. Distinct response patterns were found in the two unimodal conditions (VV and HH), while the patterns of the cross-modal conditions (VH and HV) resembled the patterns of their response modality.

### Constant error

**Figure 3:**
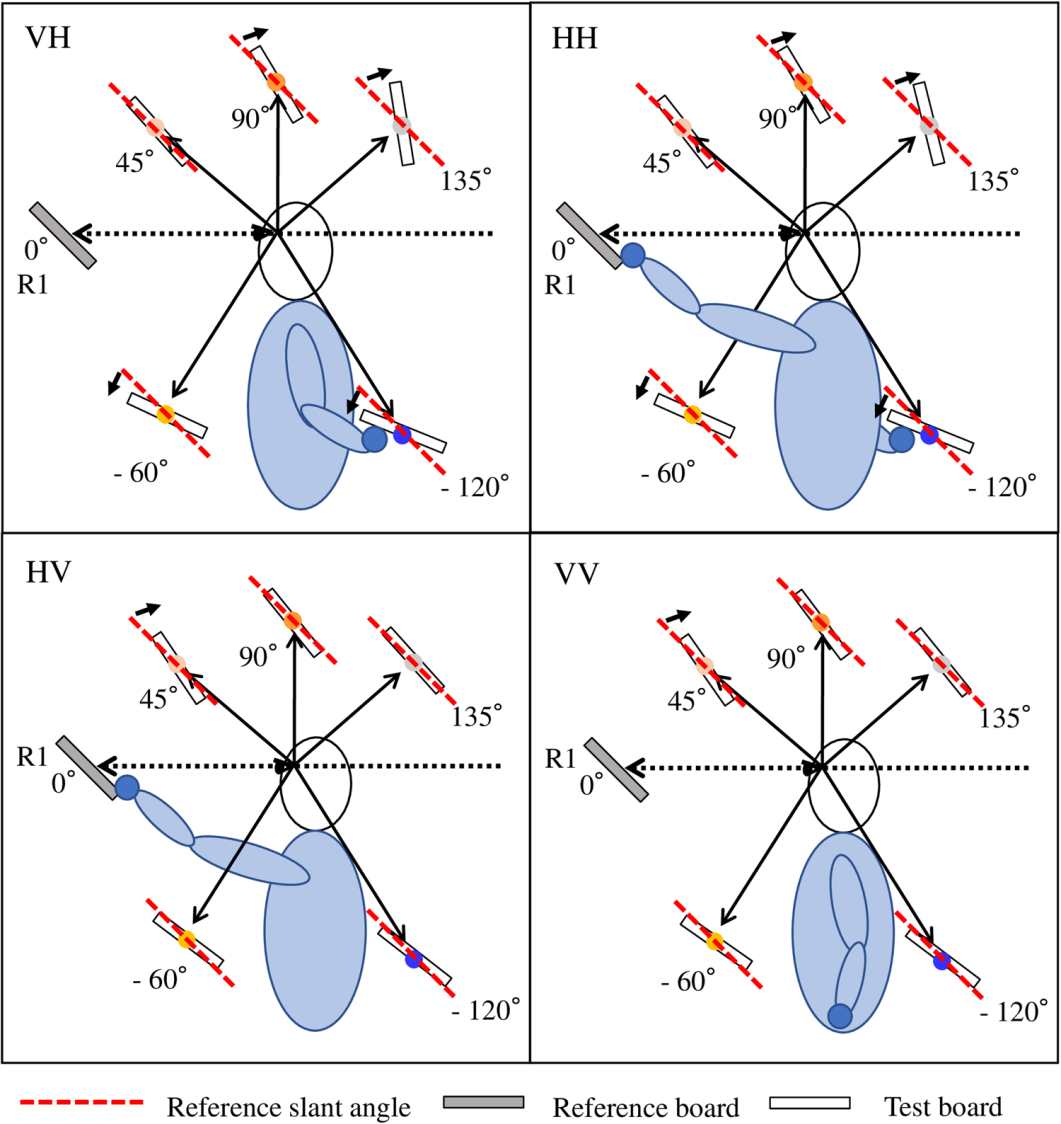
Mean responses to 45° reference angle at reference location R1 for trials at the five different test locations in the four modality conditions. Small arrows highlight the average responses, showing notable deviation from the reference angle.

**Figure 4:**
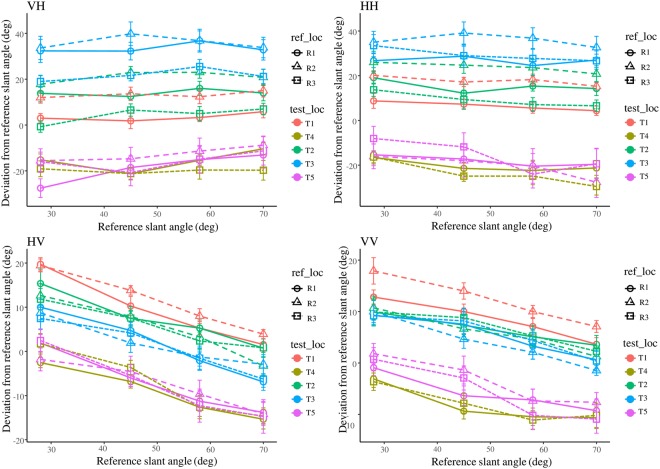
Constant errors in the four modality conditions plotted against reference slant angle. Line colour represents the test location. Line type and marker refer to the reference location. Location pairs R3T1 and R2T4 were excluded because they were overlapping or too close for the tasks. Error bars indicate the standard error of the mean across participants. The response patterns of unimodal tasks (HH and VV) were distinct, and those of cross-modal tasks (VH and HV) were not the mixture of the two patterns, but were similar to the patterns of the response modality, HH and VV, respectively.

We quantified the constant error using a signed distortion value computed as the amount the response angles were biased away from the reference slant angle. Positive values indicate a steeper angle towards the vertical axis, while negative values indicate a shallower angle towards the horizontal axis. Figure 3 depicts the mean responses of 11 participants to a 45° reference slant angle at R1 under the four modality conditions. The common tendency for all modality conditions was for the slant of the test board to be overestimated at the test locations above the reference location, and underestimated at the test locations below the reference location. The amount of deviation differed for each location condition. In the haptic response conditions (VH and HH), larger deviations were produced at test locations farther from the reference location, whereas in the visual response conditions (HV and VV) the largest deviation was found at the nearest T1 (45°) location. This difference is clearly shown in Fig. 4, which presents the mean deviations of all 208 conditions.

A three-way within-subjects analysis of variance (ANOVA) (modality, reference/test location, reference slant angle) showed significant main effects of all three factors, interactions between each pair, and among all factors (modality: F_(3,30)_ = 9.629, p < 0.0001; location: F_(12,120)_ = 48.203, p < 0.0001; angle: F_(3,30)_ = 27.929, p < 0.0001; modality * location: F_(36,360)_ = 16.298, p < 0.0001; modality * angle: F_(9,90)_ = 27.458, p < 0.0001; location * angle: F_(36,360)_ = 1.773, p = 0.005; modality * location * angle: F_(108,1080)_ = 1.474, p = 0.002). The post hoc analysis revealed no significant difference between the VH and HH (t = 0.002, p = 0.998), VV and HV (t = 1.299, p = 0.204) conditions, but significant differences between all other combinations. This finding suggests that the patterns of deviation depended on the response modality, and the target modality had only limited influence.

We identified three features that were shared by the visual response conditions (VV and HV), but distinct from those in the haptic response conditions (HH and VH).

First, the reference slant angle significantly influenced the deviations of visual response conditions (VV and HV) at almost all locations according to the post hoc analysis, and the tendency to exhibit more positive deviations in response to smaller reference slant angles (e.g., 28°) and more negative deviations for larger reference slant angles (e.g. 70°) was consistent in all location conditions (for VV and HV, we found no interaction between location and slant angle). However, this tendency was not evident in the haptic response conditions.

**Figure 5:**
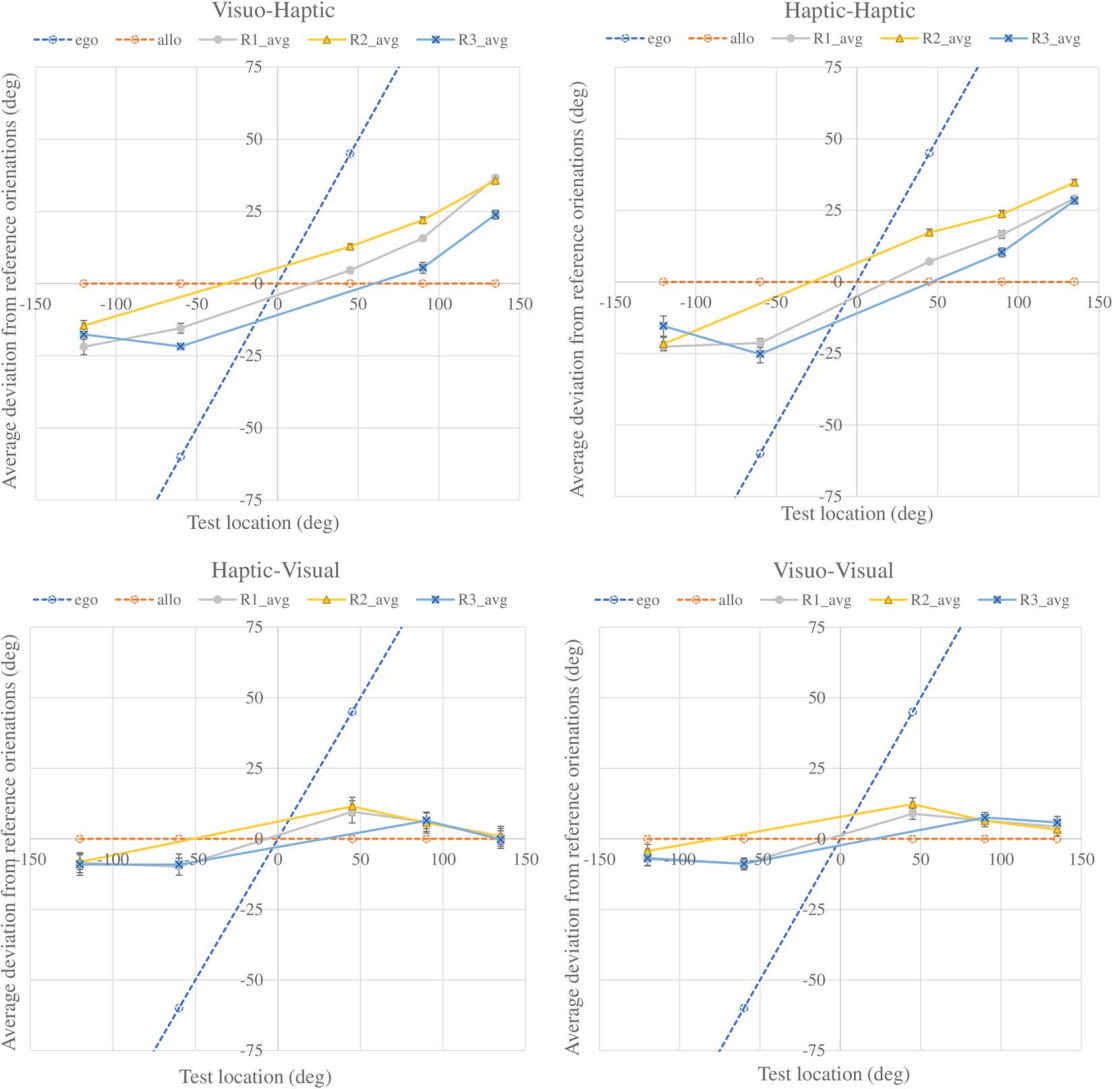
Deviation plotted as a function of test location and reference location, averaged across the four reference slant angles. Error bars indicate the standard error of the mean across participants. The dashed lines of “ego” in blue and “allo” in orange show the hypothesised deviations under a fully egocentric RF or fully allocentric RF. In the haptic response conditions (VH and HH), the deviations for different reference locations of the same test location (the position of points with the same x-axis value in the plots) were diverse, while in the visual response conditions (HV and VV), the data points of different reference locations of the same test location overlapped.

Second, the influence of reference/test location was much larger in the haptic response conditions than that in the visual response conditions. In the HH and VH conditions, the range of deviation was almost twice that in the VV and HV conditions. In Fig. 4, where the line colour refers to the test location, it can be seen that in the VV and HV conditions, lines with the same colours are close to each other, while in the HH and VH conditions, lines with different colours are intertwined. This difference indicates that the change of reference location had a stronger effect in the haptic response conditions (HH and VH) than in the visual response conditions (VV and HV), as shown in Fig. 5. The data points of different reference locations at the same test location almost overlapped in the VV and HV conditions, but varied in the HH and VH conditions.

To test statistical differences among reference/test locations, we performed multiple comparisons between all combinations of location conditions under the same modality condition and four slant angles. A t-test with Holm’s correction revealed no significant difference between all pairs of different reference locations with the same test location in the VV and HV conditions, and significant differences were only found in pairs of test locations at the opposite side of the reference location (above vs. below). However, in the HH and VH conditions, the same test revealed significant differences in many pairs of different reference locations with the same test location (p < 0.05). Interestingly, in the VH condition, t-tests analysing R1T2 vs. R2T1, R1T1 vs. R3T2, and R2T2 vs. R3T3 revealed no significant differences (p = 1.000), corresponding to the overlapping red, green and blue lines in Fig. 4. Similarly, in the HH condition, no significant difference was found in t-tests of R1T2 vs. R2T1, R1T1 vs. R3T2, and R2T2 vs. R1T3 (p = 1.000). These results suggest that the deviation in haptic response conditions was influenced by both reference and test locations. In some circumstances, different location conditions could produce the same amount of deviation. This relationship is discussed further in the “Regression models” section below.

Third, the haptic response conditions showed a stronger egocentric bias than the visual response conditions. Figure 5 shows that deviation increased monotonically with the angle of test location in the HH and VH conditions, while in the VV and HV conditions, deviation increased at T1 (45°) and decreased towards 0 at T3 (135°). We assumed that if the RF for slant matching was an allocentric RF, the deviation would be 0, and if the RF was a totally egocentric RF (e.g., arm-centred), the deviation would be the angle of the test location at least in the R1 (0°) condition, as shown in Fig. 5. An egocentric bias can be seen in the plots of haptic response conditions, but not in those of visual response conditions.

The patterns of cross-modal tasks (VH and HV) were not the combination of patterns of unimodal tasks (VV and HH). No common features were found for cross-modal conditions in terms of constant error.

**Figure 6:**
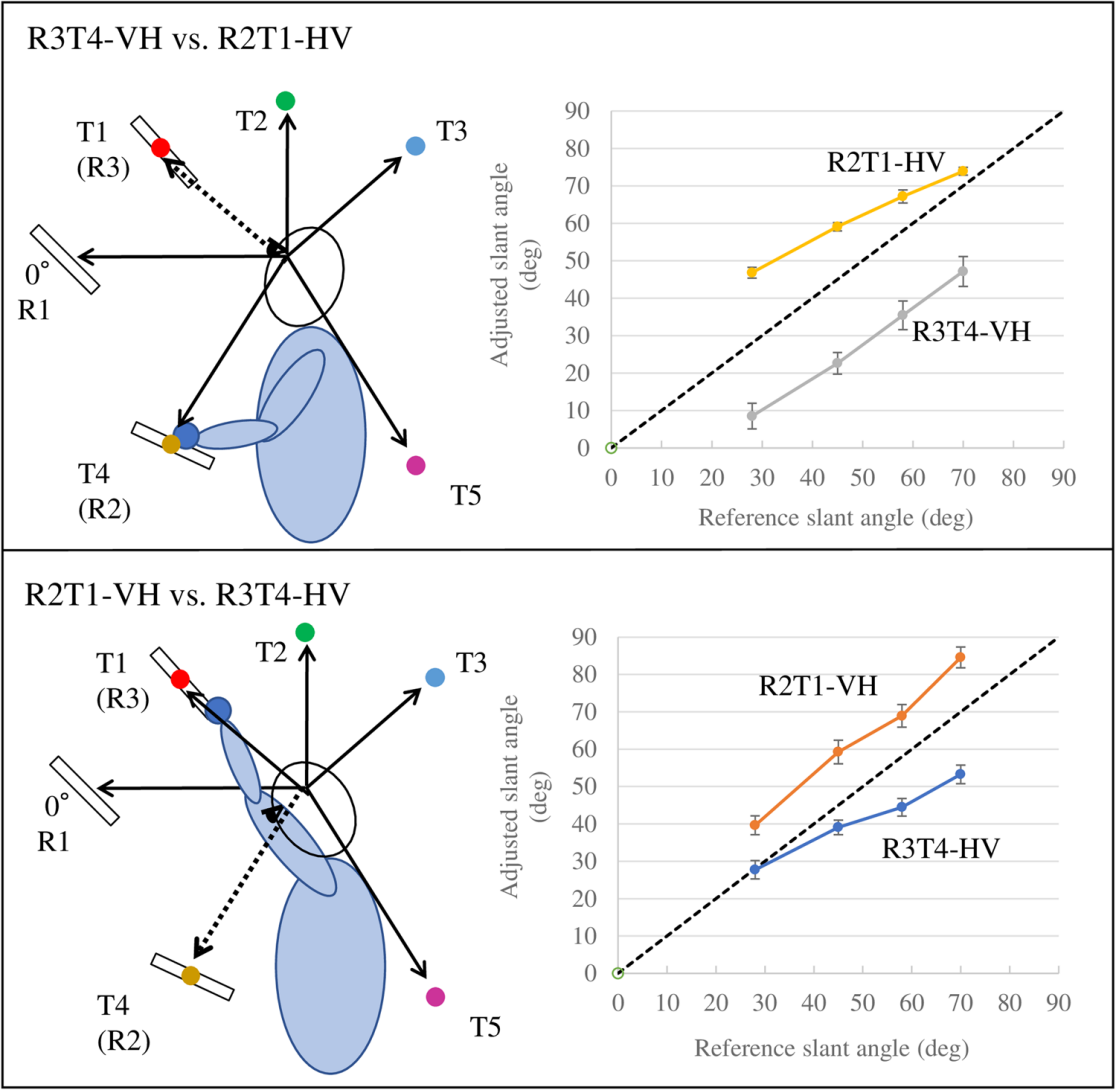
Adjusted slant angles of two pairs of conditions (R3T4-VH vs. R2T1-HV, R2T1-VH vs. R3T4-HV) plotted against reference slant angle. In each pair of conditions, participants performed the tasks with the same posture and sensory inputs, but produced significantly different results. Error bars indicate the standard error of the mean across participants.

Importantly, the same visual and somatosensory information did not produce similar responses in the two cross-modal tasks (VH and HV). When the 15° angle difference between the R2 and T4 locations was ignored, participants obtained the same sensory inputs with identical posture in two pairs of conditions (R3T4-VH vs. R2T1-HV and R2T1-VH vs. R3T4-HV) as shown in Fig. 6. Because positive deviation from the reference slant angle was produced when the test location was higher than the reference location and negative deviation was produced when the test location was lower, we excluded this factor by comparing the absolute values of deviations in the two pairs of conditions using Welch’s t-test. The slant matching results were significantly different in each pair (R3T4-VH vs. R2T1-HV: t_(71.483)_ = 4.114, p = 0.0001; R2T1-VH vs. R3T4-HV: t_(81.978)_ = 2.746, p = 0.007). These results suggest that different computations were implemented in the two cross-modal tasks (VH and HV).

### Response variability

**Figure 7:**
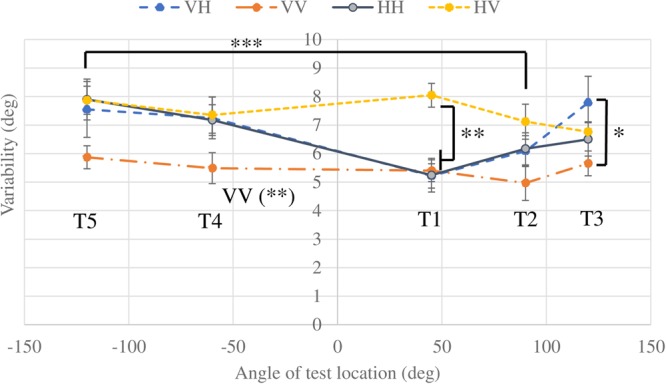
Response variability as a function of the angle of test location and modality condition averaged across three reference locations. Error bars indicate the standard error of the mean across participants. The asterisks refer to significant differences between different test location conditions (***p < 0.005) and different modality conditions (*p < 0.05, **p < 0.01) based on ANOVA and post hoc tests.

A participant’s response variability of deviation was quantified as the standard deviations (SDs) of the trial errors to the four reference slant angles under the same location condition (one of the 13 reference/test location pairs). To achieve the normal distribution required to perform an ANOVA, values of SD were transformed using the function log (SD + 1) before performing the statistical tests^19^. A two-way within-subjects ANOVA (modality, location) revealed main effects of modality and location (modality: F_(3,30)_ = 7.932, p = 0.0005; location: F_(12,120)_ = 2.651, p = 0.004). The post hoc comparisons (Ryan’s method) showed that the variability of the VV condition was significantly lower than that of other conditions (VV-HV: t_(30)_ = 4.830, p < 0.0001; VV-VH: t_(30)_ = 2.896, p = 0.007; VV-HH: t_(30)_ = 2.905, p = 0.007). In all modality conditions, location R3T5 (45°/−120°) showed significantly higher variability than location R1T2 (0°/90°) (p = 0.0012) and R3T2 (45°/90°) (p = 0.0009), indicating that the precision in the frontal space was better than that in rear space. At location R1T1 (0°/45°), the variability of the VV and VH conditions were significantly lower than that of the HV condition (HV−VV = 0.219 ± 0.050, p = 0.008; HV–VH = 0.175 ± 0.035, p = 0.003). The difference between HV and VV conditions could be the influence of transformation between RFs in the HV condition. The wrist angle at T1(45°) location might be more natural for haptic slant perception than other locations and the angle difference of R1T1 location was small so that the precision of VH condition was better than that of other locations. In the constant error section we reported that performance was more influenced by different slant angles in the HV than the VH condition. When we calculated the variability using responses to different reference slant angles, this may be another cause of the higher variability in the HV compared with the VH condition. At location R1T3 (0°/120°), the variability of VH and VV significantly differed (VH–VV = 0.195 ± 0.047, p = 0.012), which again showed the deterioration of performance in rear space for the haptic response condition. Because there was no significant effect between the same test location with different reference locations, for clarity we averaged the variability at the same test locations over different reference locations to present the results in Fig. 7. The post hoc comparisons (Ryan’s method) also revealed no significant differences among locations in VV and HV, but significant differences in HH (p = 0.037) and VH (p = 0.003). The variability was not reduced in cross-modal tasks because no redundant information was provided in our cross-modal tasks, which was distinct from the optimal integration process usually found in multisensory tasks^35–37^. The finding that the variability of cross-modal task VH was almost the same as unimodal task HH indicated that the transformation in the VH condition did not introduce a large amount of noise. In contrast, the finding that the variability of the cross-modal task HV was markedly higher than that of unimodal task VV suggested that it involved a different transformation process compared with the VH task, which introduced more noise. Because of the inevitable transformation between modalities, cross-modal tasks typically have lower precision (higher variability) compared with unimodal tasks, but the accuracy (constant error) was not significantly influenced, and stayed within a similar deviation range to the unimodal conditions of the response modality (shown in Fig. 4).

### Regression models

We quantitatively investigated the relationships between constant error (deviation *θ*_*d*_) and three independent variables (the angle of reference location *θ*_*ref_loc*_, the angle of test location *θ*_*test_loc*_, the reference slant angle *θ*_*ref_ang*_) under different modality conditions using multiple linear regression. Table 1 shows the regression equation and results. Consistent with our statistical analysis, the best predictor for the VH condition was the reference and test location, and the reference slant angle had no influence. However, the reference slant angle was an important predictor for the HV condition, along with the test location. For unimodal tasks, all three variables played roles in the fitting model. We found better predictions in the haptic response conditions than in the visual response conditions. The R-square values in VH and HH were markedly higher: 0.906 (VH) and 0.912 (HH) vs. 0.719 (HV) and 0.715 (VV), suggesting that other factors influencing the performance of HV and VV were not included in our experimental design.

**Table 1:** Multiple linear regression results.

Model	*θ*_*d*_ = *a* + *bθ*_*ref_loc*_ + *cθ*_*test_loc*_ + *dθ*_*ref_ang*_						
Modality	Coefficients		t	Pr	F_(3,48)_	p	Adjusted R^2^
VH	*a*	−3.738	−1.325	0.191	165.0	<**0**.**0001**	0.906
	*b*	−0.135	−5.693	<**0**.**0001**			
	*c*	0.176	20.722	<**0**.**0001**			
	*d*	0.098	1.830	0.073			
HH	*a*	8.205	2.740	**0**.**009**	176.4	<**0**.**0001**	0.912
	*b*	−0.104	−4.130	**0**.**0001**			
	*c*	0.197	21.923	<**0**.**0001**			
	*d*	−0.419	−2.628	**0**.**011**			
HV	*a*	17.138	7.568	<**0**.**0001**	44.5	<**0**.**0001**	0.719
	*b*	−0.037	−1.941	0.058			
	*c*	0.051	7.516	<**0**.**0001**			
	*d*	−0.358	−8.337	<**0**.**0001**			
VV	*a*	11.435	5.930	<**0**.**0001**	43.7	<**0**.**0001**	0.715
	*b*	−0.041	−2.494	**0**.**016**			
	*c*	0.053	9.156	<**0**.**0001**			
	*d*	−0.217	−5.933	<**0**.**0001**			

(Dependent variable: deviation *θ*_*d*_; independent variables: the angle of reference location *θ*_*ref_loc*_, the angle of test location *θ*_*test_loc*_, the reference slant angle *θ*_*ref_ang*_).

**Figure 8:**
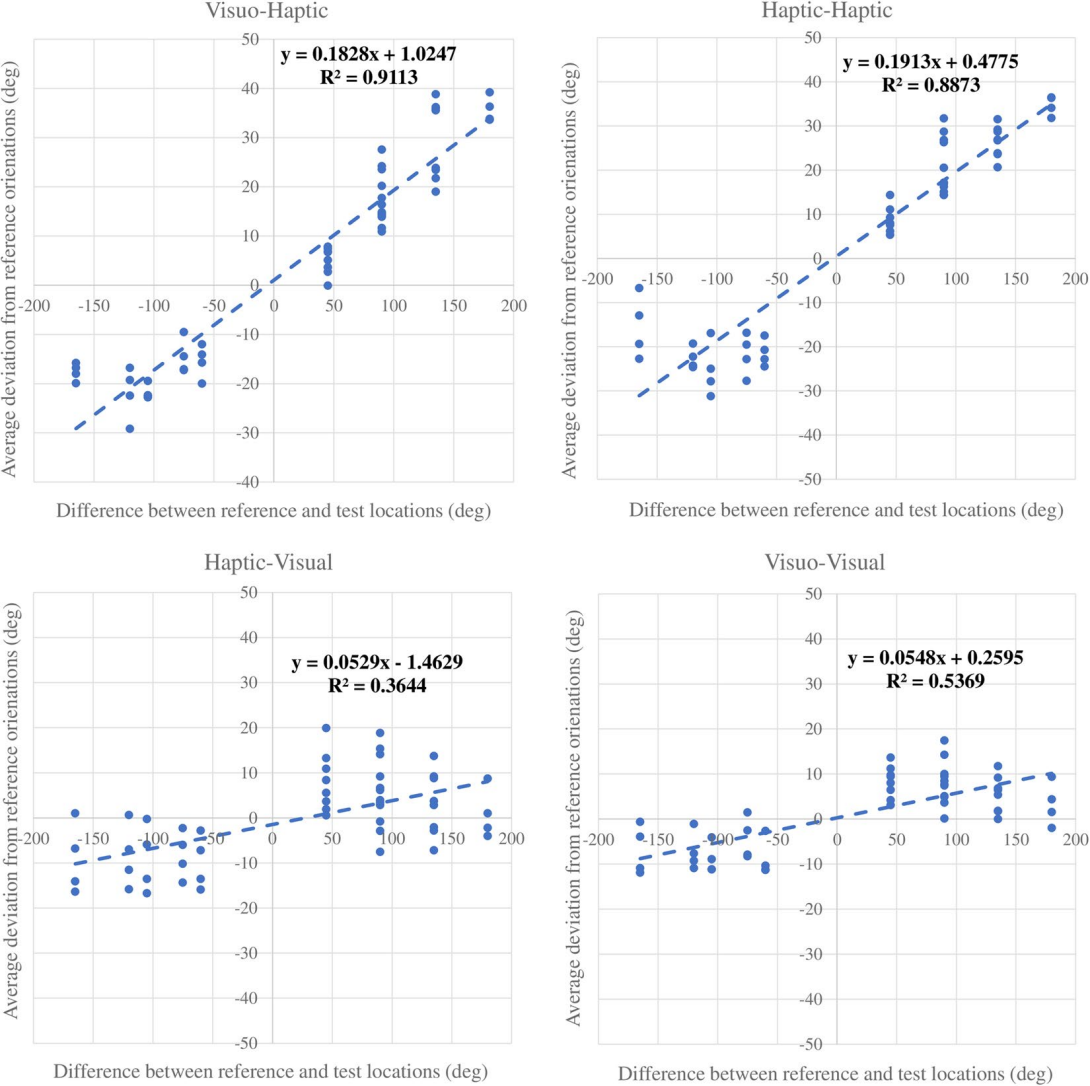
Regressions of constant errors on the angular difference between the reference and test locations. The four scatterplots present observed mean deviations across participants for all conditions. Blue dashed lines show the linear regression results taking the angular difference between the reference and test locations as the independent variable.

According to the coefficients in the fitting model, we combined the two location variables into one: the angular difference between the reference and test locations (*θ*_*diff*_ = *θ*_*test_loc*_ − *θ*_*ref_loc*_). The new linear model (*θ*_*d*_ = *kθ*_*diff*_ + *l*, where *k* and *l* are the coefficients) was able to produce high-quality predictions in the VH and HH conditions (R-square: VH: 0.911; HH: 0.887). However, for the HV and VV conditions, this variable could not generate good predictions (R-square: HV: 0.364; VV: 0.537) (shown in Fig. 8). This regression result confirmed our observation of the overlapping red, green and blue lines in the plots of VH and HH in Fig. 4, which suggested that in haptic response tasks, different reference/test location conditions could produce similar amount of deviation if the two conditions had the same angular differences between the reference and test locations.

## Discussion

We assumed that the similarity between patterns of constant error in cross-modal conditions and unimodal conditions could reflect the RF used for comparison or the relative weighting of RFs. If the pattern in the VH condition was similar to that of the VV condition, multiple sources of sensory information may be transformed to visual RF to perform the comparison (Fig. 1b), or visual RF could be weighted more heavily in this process (Fig. 1d). If the pattern in the VH condition was similar to that of the HH condition, haptic RF could have played a more important role in performing the comparison (Fig. 1a or Fig. 1d). If there was integration of multiple RF signals, the change of response variability in cross-modal tasks could provide further information about the process according to the maximum likelihood principle (MLP)^35,36^. If the patterns in the VH and HV conditions were similar but different from either the VV or HH conditions, a common RF might be introduced in the process (Fig. 1c).

The current results revealed systematic biases in slant matching tasks. Moreover, we found that the patterns of these biases depended on response modality, rather than target modality (i.e., the HH and VH conditions had similar patterns of constant error, as did the VV and HV conditions; Fig. 4). Furthermore, the patterns in the VH and HV conditions were significantly different. The results indicated that in the four models depicted in Fig. 1, transforming sensory information to the RF of target modality (Fig. 1b) or a common RF (Fig. 1c) for comparison was unlikely to have occurred in cross-modal tasks. The CNS either performed cross-modal tasks in the RF of response modality (Fig. 1a) or conducted comparison in RFs of both modalities then integrated them as a weighted average for adjustment (Fig. 1d). If it is the latter case of using multiple RFs (Fig. 1d), the variability of cross-modal conditions (VH and HV) should be similar because they both combined the results in the RFs of the response modality and target modality to make the parallelity judgment. However, in the current study, response variability was significantly different for VH and HV at test location T1 (45°) in Fig. 7. These results suggest that, in cross-modal slant matching tasks, multi-sensory information may have been transformed to the response modality and compared in the corresponding RF (Fig. 1a) (i.e., the processing flow in the VH condition was opposite with respect to the HV condition).

Although the CNS can transform sensory signals from the response modality to the target modality, particularly when the target modality (V) has much lower variance than the response modality (H) in the VH condition, our results suggested that the sensory processing flow was not determined solely by the reliability or availability of signals, but was influenced by the computations required for the whole task execution. The processing flow shown in Fig. 1a with only one transformation is the simplest of the four hypothesised models. In the other three models, the results of the comparison must be transformed back to the response modality for slant adjustment. Conducting most computations in the response modality would not require this step, and could potentially reduce the metabolic cost and noise of multiple transformations. In daily life, this more efficient, less energy-consuming strategy, performing cross-modal tasks in the RF of the response modality, may be the default mechanism. It remains possible that under certain circumstances or after training, participants might be able to perform cross-modal tasks by optimally integrating all sensory information (e.g., the model in Fig. 1d). However, in the current experiment, when naive participants conducted the tasks in a natural way, we found that the matching performance was not optimised in VH and HV conditions (i.e., participants did not exhibit the same level of performance with the same amount of information, as depicted in Fig. 6). The current results revealed that the model using the RF of the response modality in Fig. 1a was more plausible than the integration model in Fig. 1d. Recent neurophysiological investigations in monkeys have suggested that during reaching and grasping tasks, RF transformations occur instantaneously in area 5d as soon as the target of movement is presented^38^, and the RFs of single neurons in V6A remain stable in the subsequent planning and execution phases^39,40^. These findings support the model depicted in Fig. 1a.

The results in the unimodal conditions (HH and VV) were consistent with previous studies of haptic parallelity^34,41^ and visual slant perception^26,28^ in which the haptic bias reflected the strong influence of an egocentric RF, and the visual bias showed systematic overestimation of steepness when the test location was above the reference location. Similar to the findings of Volcic, Kappers and Koenderink^31^, the deviation of HH condition increased with the distance between the two surfaces. We also obtained a similar result to that reported by Coleman and Durgin^32^, revealing that participants produced opposite matching errors in the VH condition when the haptic test board was above or below the visual reference board position. Our data further revealed that in the VH condition, the matching error was positively correlated with the difference in the angles of the reference and test locations, similar to the pattern observed in the HH condition.

Because the visual modality showed less egocentric bias in the unimodal task (VV), it was surprising that the reference location had a similarly strong effect in the VH and HH conditions. Looking at a reference surface that is closer to a touched test surface could produce better matching results than looking at a reference surface that is further away. This finding suggests that gaze direction influenced the accuracy of haptic slant matching in the VH condition in the same way the location of hands affected the accuracy in the HH condition. Replacing the haptic target with a visual target in the haptic matching task (i.e., from HH to VH) did not reduce the egocentric bias or improve the haptic matching performance per se. Similarly in visual matching tasks, the haptic target in the HV condition did not significantly reduce the accuracy compared with that of the VV condition, although the variability of the HV condition increased.

In contrast, changes in response modality had more impact on slant matching performance. The results revealed that visual responses generally produced smaller distortions, in line with previous studies reporting that visual responses reduced constant error^34,42^ in parallelity tasks. We found that visual responses did not always outperform haptic responses. For example, the performance in the VH condition at location R1T1 (0°/45°) was much better than that in the VV condition. This finding is in accord with previous studies of hill perception^26,33^ in which participants visually overestimated the steepness of a hill, while their haptic slant estimation of a visible hill was close to the real slant. However, we demonstrated that this phenomenon did not occur in conditions where the reference surface and the test surface were more than 45° apart. When the angle difference was large, large distortions were produced while haptically matching the slant of the test surface to the visible reference. The findings of geographical slant perception studies^26,33^ thus represent a special case of slant perception in which the angle between gaze direction and hand location is small.

In summary, in the current study we systematically investigated the relationship between sensory modalities and RFs for cross-modal tasks in peripersonal space. The current findings demonstrated that, in cross-modal slant matching tasks, the response modality contributed more to defining the patterns of constant bias and response variability than the target modality. The performance of haptic matching to a visual target could be predicted by the angular difference between the reference and test locations, while the results of visual matching to a haptic target were influenced by the target slant angle and the test location. The features of the cross-modal VH and HV tasks were not similar, but were congruent with those of unimodal HH and VV tasks, respectively. These findings suggest that the computation for matching may be performed in the RF of the response modality.

## Methods

### Apparatus and stimulus

The stimuli were reference and test surfaces comprising two 230 mm × 200 mm (25.4° × 22.2° in visual angle) polystyrene foam boards with 7 × 7 checkerboard patterns (Fig. 1a). The reference board was mounted on a tripod, and its slant angle could be adjusted using a handle. The test board had a rod (5 mm in diameter) passing through its centre so that the board could be rotated freely. Both ends of the rod were fixed by two stands (Fig. 1a). Participants were seated on a height-adjustable chair facing straight ahead, so that all participants’ eye positions would be similar during the experiment. The slant angle was measured using a dial-type gradient scale (Blue slant 78551, Shinwa Rules).

### Procedure

Participants were asked to haptically (H) or visually (V) adjust the test board to set it physically parallel to the reference board perceived visually or haptically in 3D space. The experimental conditions comprised combinations of four slant angles of the reference board (28°, 45°, 58°, and 70°) (Fig. 2b), three reference locations (0°, −45°, and 45°) and five test locations (45°, 90°, 135°, −60°, and −120°) in the midsagittal plane (Fig. 2c), as well as four modality conditions (target-response: VV, VH, HH, HV). Some of the reference and test locations were excluded because they were overlapping or too close (i.e., 45°/45° and −45°/−60°). Thus, only 13 pairs of reference/test locations were used in the experiment. In the VV and VH conditions, the slant of the reference board was viewed binocularly, while in the HH and HV conditions the slant of the reference board was perceived by one or both hands. Before each matching trial, the test board was set to a random initial angle. In the visual response conditions (VV and HV), participants could look at the test board and give verbal instructions to the experimenter to rotate it until they judged that the two surfaces were parallel. In the VH condition, participants manipulated the test board with one or both hands to adjust it until it was parallel to the visible reference board. In the HH condition, participants touched the reference board with one hand and manipulated the test boards with the other hand so that the influence of memory could be ignored. When the participants finished their adjustment, they informed the experimenter, who measured and recorded the slant of the test board. In the HH condition, participants were asked to wear an eye mask during the task. In the VV, HV and VH conditions, when both the reference and test locations were in the frontal plane, participants were asked to wear goggles to limit their visual field. When perceiving or making adjustments haptically, participants were not permitted to look at their hands, but could use both hands freely. The experiment consisted of 208 conditions (13 reference-test location pairs × 4 slant angles × 4 modality conditions). The reference location was tested in a fixed order: 0°, −45° and 45°. The test location was presented in a counterbalanced order assigned randomly to participants. In each session for one reference-test location pair, the slant angles and modality conditions were tested in a pseudo-random order. Because of the time for setting and measuring, it took approximately 4 hours for each participant to complete the experiment, which was conducted over 2 days (2 hours per day).

### Participants

Eleven participants (seven females, mean age ± SD: 24 ± 3.2 years) were recruited for our experiment. All participants were right-handed, and had normal or corrected to normal vision, normal hearing, and reported no known motor deficits. They were paid for their participation (6,000 JPY per 2 hours; a total of 12,000 JPY) and were naïve to the purpose of the experiment. All experiments, including any relevant details, were performed in accordance with the relevant guidelines and regulations approved by the ethics committee of the National Institute of Information and Communications Technology (NICT). Written informed consent was obtained from all 11 participants prior to the experiments.

### Statistical tests

We analysed the data in terms of deviations from parallelity in aligning the test board in relation to the reference board. We used a within-subjects ANOVA to test the effects of target/response modality, reference/test location and reference slant angle on the deviation under various conditions (208 responses). The constant error in each condition was computed as signed error, where positive errors correspond to steeper slant angle. We quantified each participant’s variability of deviation as the standard deviations (SDs) of trial errors to the four reference slant angles under the same location condition (one of the 13 reference/test location pairs). To achieve the normal distribution required to perform an ANOVA, values of mean SD were transformed using the function log(SD + 1) before performing the statistical tests^17^. We used Ryan’s procedure for *post hoc* tests, and performed t-tests with Holm’s correction for multiple comparisons.

### Data availability

All data analysed during this study are included in the Supplementary Information file.

## Electronic supplementary material

Dataset 1
